# Congenital multiple tracheal diverticula

**DOI:** 10.11604/pamj.2024.47.134.42933

**Published:** 2024-03-22

**Authors:** Ashwin Karnan

**Affiliations:** 1 Department of Respiratory Medicine, Jawaharlal Nehru Medical College, Datta Meghe Institute of Higher Education and Research, Sawangi (Meghe), Wardha, Maharashtra, India

**Keywords:** Cough, dyspnea, bronchitis, emphysema

## Image in medicine

A 30-year-old male presented with complaints of chronic productive cough, breathing difficulty and occasional dysphonia. The patient had no significant personal or family history. The chest X-ray was unremarkable. Computed tomography of the chest revealed scattered ground glass opacities with a tree-in-bud appearance in the right middle and lower lobes with multiple air-filled hypodense areas around the trachea suggestive of tracheal diverticula, which was confirmed by bronchoscopy. The patient was treated with intravenous antibiotics, mucolytics and chest physiotherapy. The patient underwent endoscopic laser cauterization and is currently on follow-up. Tracheal diverticula also known as tracheocele are outpouching from the tracheal wall. It can be single or multiple invaginations of the tracheal wall. They are common in males and are usually incidental findings where the patient is often asymptomatic. They may be congenital or acquired (either due to chronic lung disease or iatrogenic). Congenital diverticula are smaller than acquired and have the same structural anatomy as the trachea. The vast majority are located at the right posterolateral aspect of the trachea. Multidetector computed tomography is essential for diagnosis. Treatment modalities include conservative management in asymptomatic patients and fulguration, transcervical or endoscopic resection in symptomatic patients.

**Figure 1:**
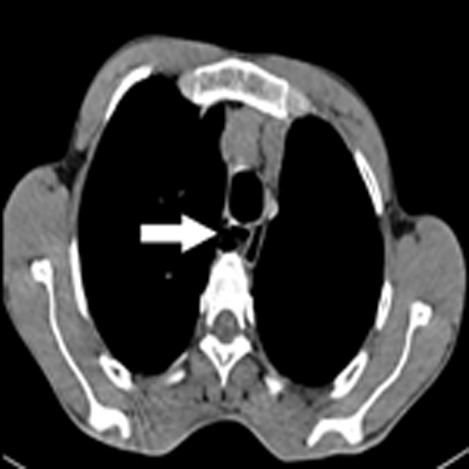
computed tomography of the chest with a white arrow showing multiple tracheal diverticula

